# Hospital and Institutional News

**Published:** 1918-12-14

**Authors:** 


### HOSPITAL AND INSTITUTIONAL NEWS.

### THE ELECTION AND INFLUENZA.

Dr. H. Kerr, the medical officer of health for Newcastle, has addressed an appeal to the agents of the local Parliamentary candidates to "restrict" on behalf of their candidates "public gatherings or personal contact between individuals" on the ground that nothing would be more likely to spread influenza than house-to-house canvassing. Dr. Kerr enforces his warning by these words: "That the gravity of the danger is not exaggerated is evident from the recent bills of mortality for the great towns in the country, and a glance at these will convince the most sceptical."

### TO THE AMERICAN DEAD IN SCOTLAND.

The American Red Cross is to place a memorial at Islay in Scotland to the troops who lost their lives in the *Tuscania* and *Otranto* disasters. Overlooking the scattered cemeteries wherein the American soldiers are buried, the site of the monument is on the Mull of Oa, "a rocky promontory 500 feet above the sea." The *American Red Cross Bulletin* has published an illustration of the memorial, which resembles a tall round tower with a conical top, and stands sixty feet high. It will be built of the local stone. The name of its designer is not stated.

### RELICS OF THE FIGHT.

The relics of H.M.S. *Vindictive*, of which the vessel's coat-of-arms was the chief, were sold by auction, at the recent Newmarket bloodstock sale, for the benefit of the Dover Red Cross. The coat-of arms eventually fetched 1,050 guineas, and fragments of wood and iron from the ship 163. We wonder what has been preserved for the United Service Museum, and the War Museum also.

### GOOD THINGS AT A HOSPITAL FAIR.

An amusing fact about the fashionable fair held at 16 Carlton House Terrace for the Royal Free Hospital was the "run" on the stalls which sold food: wildfowl, fruit, and things to make the rationed mouth to water. On the stall of antiques was an old medicine chest with bottles, phials, and, we hope, examples of those bleeding cups which are still to be found occasionally in the older households of America. On the American food stall, the food was to be noted for its weight—hundredweights of it, in fact—and the Canadian stall was also heavy with eatables. We are all esurient nowadays, and such stalls perhaps can provide dainties not otherwise to be found for Christmas.

### TEN-YEAR ANNUITIES FOR HOSPITALS.

To the annual subscriber retired from active work whom the high rate of income-tax has induced to curtail his gifts, Mr. Roderick M. Macgregor has made the following suggestion. Why not, he urged in the *Glasgow Herald*, release now that sum appointed for a legacy and purchase with it for the Glasgow Royal Infirmary "a guaranteed ten-year annuity, payable direct for the next ten years certain, and continuing thereafter" so long as the donor shall live. "The older the donors are the more valuable will be their action." True; but do old gentlemen favour the alteration of their wills?

### THE ORGANISATION OF VOLUNTARY WORKERS.

The National Council for Combating Venereal Diseases, at its Liverpool branch, lately organised a conference of women social workers, to suggest to social workers how each in her own sphere may make the best possible use of the influence she possesses over those among whom she works, to secure for the coming generation freedom from the dangers of venereal diseases. The chairman, Mrs. Herbert Rathbone, gave an address on the history of the Liverpool campaign against venereal diseases. "Local facilities for the treatment of venereal diseases" were described by Dr. Edna Mawson, City Hospital, Fazakerley, and "The social and moral aspect of the problem" by Miss Cowlin, organiser, Liverpool Women Patrols. "The problem as it affects infants and children, adolescents, the mentally defective, and inmates of Poor-Law institutions, hospitals, and prisons" was discussed by various expert speakers, and at the second session "The responsibility of women in reference to some problems of national health" was the chief topic examined. It is hoped to make the conference an annual event.

### WESTERN INFIRMARY, GLASGOW.

The report of this institution always has a special interest on account of the clear and exhaustive manner in which the accounts are presented. The financial year ends on October 31, and the deficit caused by the excess of ordinary expenditure over ordinary income was £17,853. Large as this sum is, it is not nearly so large as the deficit in the previous year, which amounted to £21,652. The contributions from subscribers show an increase of £5,227, and the ordinary income from all sources exceeded that of the previous year by £13,741. No better evidence than these figures could be afforded of the appreciation of the excellent administration of the Western Infirmary by the people of Glasgow and Scotland generally. The feature of the year's report was, however, the generous gift of Lord and Lady Newlands of £26,600 5 per cent. War Stock to complete the endowment of the Lady Hozier Home, Lanark. This is an integral part of the Western Infirmary. In 1914 these noble donors intimated their wish to commemorate the visit to Glasgow of Their Majesties by a gift of £25,000. The interest on gilt-edged securities, through the war, was materially depreciated, so Lord and Lady Newlands have paid the interest on £25,000 each year since 1914, and now complete their gift by the transfer of the War Stock mentioned. Always alert to the progressive development of our great hospitals, the authorities of the Western Infirmary have wisely made a new departure by establishing a school for massage, medical electricity, and

Swedish remedial exercises. This is the first school of massage created in Scotland in association with a general hospital. The demand which existed for this school of massage is shown indisputably from the fact that 319 candidates applied for training in January; of whom fifty-one have been enrolled. The Incorporated Society of Trained Masseuses held their first examination in Scotland in July 1918, and amongst the candidates who presented themselves were thirteen from the Western Infirmary, all of whom gained their certificate.

### A MEDICAL MISSION EXHIBITION.

At last the London Diocesan Board of Missions appears to have awakened to the need for arousing public interest in medical missions, the Cinderellas of the mission field for news of whom we have often pleaded, and generally in vain. The Conference of British Missionary Societies has agreed to join an inter-denominational committee, with the Bishop of Stepney for its head. The committee is to organise a Medical Missionary Exhibition next June at the public hall attached to St. Martin's-in-the-Fields, which, as Londoners know, has an open space attached to it. The aims of the exhibition include an attempt to secure for medical missions the "support and life service" of doctors and nurses who have been on duty through the war.

### SECTIONAL SUBSCRIPTIONS AT WORCESTER.

It must be some satisfaction to the organisers of Newcastle and Cardiff to see their success quoted in centres which at a comparatively late hour have decided to arrange sectional subscriptions. Worcester is one of these, and it is a popular mistake to suppose that cathedral cities do not have their industries. Though the new scheme is to affect these last, it is fair to remember another section of subscribers—namely, those who give an annual sum—has maintained its contribution for twenty years. There is no reason why, as at York, a farmers' fund should not be started in the outlying districts, nor, as in North Staffordshire, a separate fund from the employers. At Cambridge, Addenbrook's Hospital organised a series of regular gifts in kind from neighbouring villages, and it is common for county towns to have special collections from the shops. We mention these examples because it is never too trite to repeat that every class which a hospital serves should have its own fund for the benefit of the hospital to which each is entitled to go according to its need.

### COTTAGE HOSPITALS AND SECTIONAL SUBSCRIPTIONS.

One promise due to be fulfilled "after the war" is being kept by Sir W. P. Hartley, who has told the committee of Colne Cottage Hospital that he is ready to proceed with his scheme for a new hospital for the town, which has been suspended since October 1914. His plan was to build and equip a hospital at a cost of £30,000, provided that the workpeople would agree to a system of weekly contributions, at the rate of one penny a week from all earning over 10s., for its maintenance. If the trade union leaders agree, as is hoped, the hospital will be built near the Hartley Homes, and will accommodate twenty-four beds and a children's ward. The present cottage hospital at Colne, which was also built by Sir William Hartley, will still continue its work.

### A COTTAGE HOSPITAL FOR STONE?

At last, we understand, a serious effort is to be made to provide Stone, in Staffordshire, with the cottage hospital which it has long hoped for. The medical officer has often urged the matter, for, as things are, accidents have had to be taken as far as Stoke or Stafford. The Nursing Association has also tentatively pleaded. Now the Urban District Council has tried to set the ball rolling. The only unfavourable fact is that no public-spirited man of position seems to have taken the matter up, and made himself a voluntary sponsor for the scheme. Is there really no such person?

### THE STRIKE IN LLOYD'S TOBACCO FACTORY.

This strike reveals a curious mental attitude of the wage-earners in this factory. Their action may be most disastrous to the interests of men partially disabled by wounds. The management of the factory offered trial to two wounded men, and after trial found that the men did not suit their requirements by reason of their wounded condition and discharged them. Immediately the other workers demanded the reinstatement of the wounded men, and failing to obtain their demand went out on strike. There seems no reason to suppose that a strike would have occurred if Messrs. Lloyd had refused to give a chance to any wounded men. If they had not been sympathetic to the wounded the question of discharging wounded would not have arisen, and work in the factory would have gone on in routine fashion. The action of the strikers is certain to make many employers hesitate to give chances to the wounded for fear of similar happenings in their works, and so the hopes of employment for the wounded will be by so much lessened. The curious vicarious charity of the strikers is expressed by their spokesman, "If your industry cannot carry these men on its back, God help it," or words to that effect. It does not seem to have struck the speaker that if they really feel charitable he and his fellows could easily carry these two men on their backs. A voluntary income-tax on the pay of all the strikers of one penny in the pound sterling would keep the men they pity so much in affluence when the sum thus obtained was added to their pensions. This would surely be a better course for the strikers to take than to use the strike weapon in an attempt to wring unwilling charity from their employers. The strike is rightly employed in last resort to get a fair share of the profits they earn for the workers who earn them. It is wrongly used if used to compel employers to pay men more than their services are worth—that is as if a man should use a useful tool for a criminal purpose.

### WOUND PENSION AND TAXATION.

One of the paragraphs of the Silver Badge Men's political programme demands that all service and disability pensions shall be free from income tax. The demand shows a fundamental misunderstanding of the duties of a citizen and the meaning of taxation. Citizens share certain advantages of social life in common. Each should be proud to pay his share in accordance with his means. A man should be ashamed to shirk his share and to put it off his own shoulders on to the shoulders of his fellow-citizens, for someone must pay. There is no reason why the man who has denied himself and saved from the gains of a small business, or from his wages, and so put by enough for a small income in his old age, should be taxed on unearned income whilst the pensioner, whose pension, rightly considered, is a compulsory saving, should enjoy the same income without deduction of taxes. The Silver Badge Men would repudiate with scorn charitable doles, but can they not see that legislation such as this they ask for is compelling charity from men many of them no better off than themselves? If they persist in a demand to have, free of all cost to themselves, the protection of the Navy and the Army, the services of the Civil servants, and all that the community provides for its members by taxation, they will lay themselves open to comparison with those fellows who hang about bars and canteens and take a drink from any and all who will stand treat, but never, if they can help it, put hand in pocket to stand treat in return. A citizen should be as proud to pay his share of taxes as his share of drinks, and as ashamed to avoid payment of them as to shirk his turn to stand the round. The Silver Badge Men will be well advised to expunge this item of their programme.

### THE FUTURE OF THE SQUARES.

Is there any ground for the anxiety which has been expressed that London will lose its squares? We do not know, but it is odd that Mr. E. B. Chancellor, who lately praised their beauties before the Royal Society of Arts, when he said that they "were likely to become the prey of speculative builders," at the end of this very lecture also praised the work of the London County Council in preserving open spaces for the general good. Are not, then, squares technically open spaces? How is the speculative builder to gather them into his profane hand? Frankly, a no less anxious thought is inspired by the humane suggestion of another person that the squares should be thrown open to the children. We fear that this throwing open of private places, even to children, would end in there being no private places at all. Open spaces are, or should be, part of the new housing schemes, and the article in The Hospital on "Co-operative Houses," which proposed quadrangular buildings, would also place a series of open courts or "quads" throughout the slums. Is the fear justified? If so, is not our scheme the better of the two?

### PUBLICITY AND MEDICAL OFFICERS OF HEALTH

When National Health Week was imposed upon the country by personalities of whose existence an amusing tale could be told, a medical officer of health complained to us of the immense amount of work, of a purely business kind, which was thrust on him to the detriment of his proper duties. His case against Health Week was another matter. What, then, will he and his colleagues say to the cinematograph films which the Local Government Board is inviting them to grace with their presence—and, no doubt, a lecture? These are to illustrate under a popular title the best precautions to be taken against influenza. They are to point out that one should, if possible, "keep fit," and further, avoid excessive drinking. They are to explain that the handkerchief should be placed in front of the nose and mouth when sneezing and coughing. We do not quarrel with these recommendations on medical grounds. We do not even think them unnecessary. But must the medical officer of health be drawn from his quiet work and varied duties to grace the cinemas where these films are being performed? If the films are worth anything, will not the moral be obvious without the presence of the medical officer of health?

### JEWS AND THE JERUSALEM EYE HOSPITAL.

British hospitals in distant lands usually maintain such a jealous obscurity that it is pleasant to see that the Ophthalmic Hospital of St. John in Jerusalem appeals for assistance to rebuild. It is the only hospital of its class in Palestine, where eye diseases are epidemic, but having been used by the Turks as an ammunition store, was partly blown up by them on their retreat. The hospital is maintained by the Order of St. John in England, and the Order now hopes to secure an endowment of £50,000 to maintain and extend the work. Such a fund would help to mark the liberation of Palestine from Turkish rule, which has endured for over six hundred years. The new hospital which would be created would also mark the recovery of Jerusalem for Christendom. It seems to us that an appeal should be made particularly to the Jews, especially those who are interested in the Zionist movement. Though our holy places may mean little to them, not only is Jerusalem their city, but their return to Palestine, if it comes about, will be the fruit of the restoration of Palestine to Christendom—an odd situation regarding which everyone, except the Turk, may well feel pleased.

### THE ACT OF 1864.

Having lately resigned, at the age of sixty-six, after twenty-six years' completed service as housemaid (River Hospitals and Ambulance Service), Miss Louisa Stone has been awarded by the Metropolitan Asylums Board an annual allowance of £19 6s. Having contracted out of the Poor Law Officers' Superannuation Act, 1896, Miss Stone has been given the benefit of the power conferred on the Board by the Act of 1864 to award an annual allowance, subject to the approval of the Local

Government Board. We hope the Asylums Board will raise its minimum scale of small annual allowances to the equivalent of 10s. per week.

### IN DEFENCE OF THE GUARDIANS.

Mr. A. P. Stanwell Smith, Clerk to the Southwark Board of Guardians, has prepared a statement in defence of the Boards of Guardians, in which he points out that their work was constantly being developed, and that during the past ten years not only have they rendered invaluable service, but during the war have had to deal with a mass of new Orders issued by the Local Government Board. The London Guardians house about 70 per cent. of the chargeable poor of the metropolis. Practically every board had a special infirmary for the sick, which, Mr. Smith claims (?), in equipment and staff was equal to the best type of general hospital. These infirmaries were not only of great value in receiving ordinary accidents, but admission was frequently applied for and allowed to persons who did not come within the category of "destitute." In fact, he states, the best Poor-Law infirmaries were rapidly assuming a position with the general public as municipal institutions in the same way as public libraries and parks. They also formed valuable training-schools for nurses. Only twenty years had elapsed since an Order was issued prohibiting the nursing of patients by inmates. This was one instance of the evolution of the Poor Law.

### WAR WORK OF THE INFIRMARIES.

The institutions of Boards of Guardians in London, Mr. Smith adds, were found invaluable after the outbreak of war. Guardians willingly handed over their infirmaries as military hospitals, completely equipped and staffed, and supplied many thousands of beds, and the War Office were glad to take advantage of the Guardians' experience in continuing their management on its behalf. Other London institutions had been used for Belgian refugees and interned aliens, and the Guardians and their officers had readily adapted themselves to the change. Dealing with the work of the Metropolitan Asylums Board, Mr. Smith points out that the Board's twenty-seven institutions were very efficiently managed, and bore comparison with the best of their class. Mr. Smith also states that in any drastic administrative change not only would the poor suffer, but the cost of administration, both central and local, would be immensely increased. Though we have argued the matter from a point of view different from that of Mr. Stanwell Smith, it is only fair to hear the defence of the Guardians by one of their capable officers.

### SCHOOLS FOR CONSUMPTIVE CHILDREN.

A big scheme for consumptive school children has been prepared by the London County Council Education Committee, which has had evidence that there are 2,000 children definitely notified as tuberculous, and another 2,000 requiring open-air treatment. The idea is to accommodate 2,000 in twenty suburban schools, near tram routes. The Council will pay fares and also provide meals in cases of necessity. The committee preferred to disused army huts, private houses with large grounds. For each school a head teacher, a nurse, and an assistant teacher for each twenty-five children would be required, and also a cook and helper. The capital cost of such a scheme is expected to be over £100,000, and the maintenance about £58,000 a year.

### MEDICAL TREATMENT BY MIDWIVES.

The large number of cases of ophthalmia neonatorum and the difficulty in obtaining the necessary medical attention has led the Durham county medical officer of health to ask the secretary of the Central Midwives Board whether the Board would object to the County Council providing practising midwives, free of charge, with supplies of collosum argentum. The secretary has replied that the Board, whilst designedly refraining from recommending in the rules the use of any specific drug for the treatment of the eyes of newly-born infants, sees no objection to the course suggested. The medical officer, who accordingly seeks authority to supply the drug, states that from inquiries he has made he finds the preparation is non-irritating, and in most cases is effective.

### TRAINING "HOME HELPS."

The Durham County inspector of midwives reports that the essential need of the moment is the training and establishing of "home helps." Many of the patients attended by midwives have no responsible person to carry on the work of the home during the lying-in period. Certainly the women do all they can for each other at this time, but it is not an uncommon thing for the patient to do the work of the home daily, returning to bed only for the time of the midwife's visit. This state of affairs ought to be made impossible, for surely a mother is justified in claiming absolute freedom from all domestic responsibility during the puerperium. Almost daily the midwife sees the necessity for this class of help, without which her patient does not get a proper chance of complete recovery.

### THIS WEEK'S DRUG MARKET.

Generally speaking, the slow, downward tendency in prices continues, but in very few instances is there any marked reduction in value, and in no case is there anything in the nature of a sensational drop. Honey is lower in price, and with the promise of increased supplies of sugar we may expect to see a further decline in saccharin, and, in fact, in everything sweet. Isinglass is cheaper. Menthol and Japanese peppermint oil are hanging fire, but quotations are nominally unchanged. Permanganate of potash promises to be in better supply before long, and prices have a downward tendency. A good business has been done in salicylate of soda. Barbitone is lower in price, as also is chloral hydrate. Thymol is offered at rather lower prices. A good business continues to be done in eucalyptus oil at firmly maintained prices.

